# Environment vs. Plant Ontogeny: Arthropod Herbivory Patterns on European Beech Leaves along the Vertical Gradient of Temperate Forests in Central Germany

**DOI:** 10.3390/insects9010009

**Published:** 2018-01-26

**Authors:** Stephanie Stiegel, Jasmin Mantilla-Contreras

**Affiliations:** Ecology and Environmental Education Group, Institute of Biology and Chemistry, University of Hildesheim, 31141 Hildesheim, Germany

**Keywords:** adult trees, feeding guilds, feeding traces, *Fagus sylvatica*, forest layer, juvenile trees, leaf traits, microclimate

## Abstract

Environmental and leaf trait effects on herbivory are supposed to vary among different feeding guilds. Herbivores also show variability in their preferences for plant ontogenetic stages. Along the vertical forest gradient, environmental conditions change, and trees represent juvenile and adult individuals in the understorey and canopy, respectively. This study was conducted in ten forests sites in Central Germany for the enrichment of canopy research in temperate forests. Arthropod herbivory of different feeding traces was surveyed on leaves of *Fagus sylvatica* Linnaeus (European beech; Fagaceae) in three strata. Effects of microclimate, leaf traits, and plant ontogenetic stage were analyzed as determining parameters for herbivory. The highest herbivory was caused by exophagous feeding traces. Herbivore attack levels varied along the vertical forest gradient for most feeding traces with distinct patterns. If differences of herbivory levels were present, they only occurred between juvenile and adult *F. sylvatica* individuals, but not between the lower and upper canopy. In contrast, differences of microclimate and important leaf traits were present between the lower and upper canopy. In conclusion, the plant ontogenetic stage had a stronger effect on herbivory than microclimate or leaf traits along the vertical forest gradient.

## 1. Introduction

Arthropod herbivores influence the structure and functioning of plant diversity and ecosystem processes [1,2], with different effects depending on the feeding guild. Effects of leaf-chewing insects on ecosystems include influencing the composition and productivity of plant communities, as well as carbon and nutrient cycling (reviewed by [3]). Sap-feeding insects significantly reduce plant growth, reproduction, and photosynthesis (reviewed by [4]), which is important for forest ecosystems. To date, most studies about herbivory in canopies have been published for tropical forests, whereas temperate forests have received less attention. However, temperate deciduous forests reveal an uneven vertical distribution of arthropod communities in different strata [5,6]. Along this vertical gradient of temperate forest stands, environmental changes occur with increasing temperature and decreasing air humidity from understorey to upper canopies [5,7]. Spatio-temporal changes of the environment are expected to alter interactions between plants and herbivores [8]. Nonetheless, ontogenetic changes are also present along the vertical forest gradient since juvenile and adult trees occupy the understorey and canopies, respectively. Insect herbivores show a variability in their preferences for plant ontogenetic stages [9,10]. Varying frequencies of insect herbivores have been documented for some feeding guilds being more abundant either on saplings or mature plants [11,12]. Possible causes for this variation are differences in plant chemistry, leaf palatability, and local microclimate [13]. These parameters are connected to the development of plants, which can be generally categorized into ontogenetic and physiological or environmental processes [14].

Ontogenetic processes on plant development arise from alterations in plant meristem gene expression [15]. The changes in vegetative structure are widespread and occur across whole plant gradients [16]. Many plant traits, including those involved in defences against herbivores, vary between different plant ontogenetic stages. Variations among plant ontogenetic stages have been found amongst others in leaf toughness [10,17] and chemical defences such as phenolics [18,19,20]. Boege and Marquis [21] have proposed a pattern for plant ontogenetic changes in herbivory defence and tolerance with increasing levels until reaching an optimum as plants further develop. In woody plants, chemical and physical defences increase during seedling and vegetative juvenile stages, respectively, but no differences in plant defences (physical defence traits and secondary chemistry) are found between juvenile and mature individuals [22]. Furthermore, a comparison of insect herbivores revealed no preferences for juvenile or mature individuals of woody species [22]. Clearly, the great diversity of insect herbivores and feeding guilds cannot lead to one general plant ontogenetic pattern.

Environmental processes on plant development are based on factors such as shading, water, and nutrient relations, resulting in alterations of the local meristem environment. Morphological and functional leaf traits (e.g., toughness, nutrients, or defence compounds) often mediate indirect environmental effects on herbivory [23]. Based on the variation of environmental conditions, the above-ground strata of forest ecosystems represent different microclimates. Microclimatic requirements and availability of food resources within the tree canopies can reflect spatial distributions and preferences of arthropods [24]. For European beech (*Fagus sylvatica*), leaf traits change along the vertical forest gradient with unfavourable conditions (e.g., lower nitrogen content, higher carbon content, and toughness) for leaf-chewing insects in upper canopy leaves [25]. However, a variation of arthropod herbivory patterns is expected between different feeding traces, especially between different feeding guilds.

Even though knowledge about interactions of herbivory and forest ecosystems has increased during the last years, several gaps remain, and particularly for temperate forests. Most research focuses only on a single type of insect herbivore [26], or the distribution of different herbivore feeding guilds on juvenile and mature leaves [27,28]. Only few studies have surveyed the whole vertical forest gradient for herbivory research [29,30], especially including several feeding guilds [31]. Furthermore, galls have rarely been studied in upper canopies of mesic forests [32]. This study attempts to elucidate the distribution patterns of arthropod herbivory on leaves of the broad-leaved tree species *F. sylvatica*, focussing on the whole vertical forest gradient for comparisons between different microclimates, as well as between juvenile and adult *F. sylvatica* individuals. Herbivory was investigated for distinct arthropod feeding traces within four feeding guilds (leaf-chewing, sap-sucking, leaf-mining, and gall-inducing). Patterns of herbivory were analysed with respect to microclimate (temperature and relative air humidity), leaf traits (toughness, nitrogen and carbon content), and plant ontogenetic stage (juvenile and adult tree individuals) determining the main predicting parameters. Based on the knowledge that levels of herbivory differ between distinct feeding guilds, as well as within feeding guilds and species along environmental gradients, two contrasting hypotheses were tested: patterns of herbivore attacks along the vertical forest gradient are (1) caused indirectly by changing leaf traits (toughness, nutrients, and defence compounds) induced by distinct environmental conditions (temperature and air humidity); or (2) determined by the plant ontogenetic stage (juvenile and adult trees).

## 2. Materials and Methods

### 2.1. Study Site

**Figure 1:**
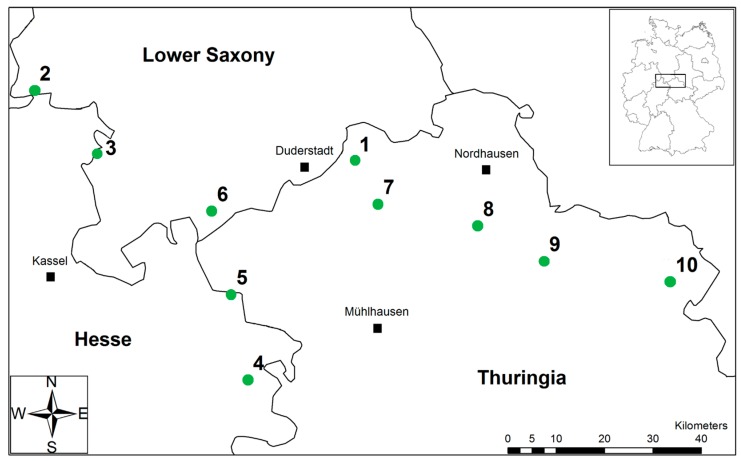
Study region in Thuringia, Lower Saxony, and Hesse with ten forest sites (green circles). Forest sites: (**1**) Winkelberg; (**2**) Tiefentals Ebene; (**3**) Klingenberg/Vaaker Berg; (**4**) Schieferstein; (**5**) Heiligenberg; (**6**) Bocksbühl; (**7**) Hubenberg; (**8**) Feuerkuppe; (**9**) Heidelberg; and (**10**) Eichleite. Original copyright: GeoBasis-DE/BKG 2015, data changed with permission from *Bundesamt für Kartographie und Geodäsie*.

The research study was conducted in the hill and mountain region of Central Germany, within the federal states Thuringia, Lower Saxony, and Hesse (Figure 1). Ten forest sites with mixed deciduous tree species were selected along a 140 km long west–east transect (altitude: 140–444 m.a.s.l.). The criteria for the forest stand selection were (i) closed canopy without major gaps; (ii) no significant presence of coniferous tree species; and (iii) a stem circumference of adult beech individuals >1 m. In the study area, mean annual temperature was about 9 °C and annual precipitation ranged from 474–874 mm (German Weather Service, reference period 1961–1990). The geological substrate of the forest sites was lower Trias sandstone, except for Bocksbühl (upper Trias sandstone), Feuerkuppe and Heidelberg (middle Triassic limestone).

Within the studied forest sites, *Fagus sylvatica* Linnaeus (European beech; Fagacecae) was the dominant broad-leaved tree species. At each of the ten forest sites, a random selection of juvenile and adult tree individuals was undertaken at two different spots resulting in 20 sample sites. Three adult individuals of *F. sylvatica* were surveyed at the lower and upper canopy (average height: 18 and 35 m, respectively), as well as three juvenile individuals of *F. sylvatica* in the understorey (average height: 1 m) at all sample sites. Lower and upper canopies of adult beech trees were accessed with rope climbing. A total of 60 adult and 60 juvenile *F. sylvatica* individuals were selected for the study.

### 2.2. Microclimate and Leaf Trait Data

Microclimate (air temperature and relative air humidity) was measured hourly with data loggers (iButton, Model DS1923, Maxim Integrated, San Jose, CA, USA). Data loggers were installed in the understorey (about 1 m height), as well as in lower and upper canopies of adult *F. sylvatica* trees (about 18 and 35 m height, respectively) at each sample site. Complete data were available from July–August 2012 for all sample sites. Average day values from sunrise to sunset (6 a.m. to 9 p.m.) were used for further analyses with temperature and air humidity. Night values for microclimate were excluded for two reasons. On the one hand, the microclimatic pattern along the vertical forest gradient is most present during daylight and can be reversed, weakened, or even disappear during the night [5,33]. On the other hand, insect herbivores show a greater activity during the day than at night [34].

Foliage material was collected in June 2012. Despite the guidelines for specific leaf area (SLA) measurements [35], foliage material had to be deep frozen due to logistical constraints during the field work until analyses of leaf traits (toughness and nutrients) and herbivory could be carried out at the university. If collected leaves could not be measured within 24 h they were stored between moist filter paper in sealed plastic bags in the freezer (−18 to −35 °C) according to the data standards protocols of the LEDA Traitbase (database of the life-history traits of Northwest European flora) [36]. The sampling period was kept as short as possible (one month) to minimize a variation of leaf traits and herbivory caused by seasonal changes (leaf age). Specific leaf area (m^2^·kg^−1^) was used as an indicator for toughness. It relates the area of a fresh leaf to its dry mass, and low SLA values are linked to structural defences [35]. Five to ten leaves were collected per tree individual in the understorey, lower, and upper canopy for analysis of SLA. The collecting time was either in the morning or the afternoon and deviated from the recommended time after sunset or before sunset [35] based on logistical constraints (access to the canopy with climbing rope). The variation of leaf sample amount was dependent on the availability of fully developed leaves without obvious symptoms of pathogens or herbivore attacks. Any petiole and all veins were considered as part of the leaf for standardised SLA [35], and were included in the SLA measurement. All frozen leaves were defrosted and scanned with a flat-bed scanner to obtain their leaf area using the computer image analysis system WinFOLIA (Régent Instruments Inc., Ville de Québec, QC, Canada). Afterwards, leaves were dried in an oven (48 h at 70 °C) and weighed to calculate SLA values (Equation (1)). For further analyses with SLA, the mean value was used for each forest layer per sample site.

(1) $$\mathrm{SLA}=\mathrm{leaf}\;\mathrm{area}\;\left(m^{2}\right)/\mathrm{leaf}\;\mathrm{dry}\;\mathrm{mass}\;\left(\mathrm{kg}\right)$$

Leaf nitrogen (N) and carbon (C) concentrations represent the total contents of N and C per unit of dry leaf mass (mg·g^−1^). Nutritional analyses were conducted with mixed samples consisting of ten fully-developed leaves per forest layer for each sample site. Leaves without petioles were dried in an oven (72 h at 60 °C) and ground afterwards. Total N and C contents were obtained with a C/N elemental analyser (Department of Plant Ecology and Ecosystem Research, University of Göttingen, Göttingen, Germany). The N and C contents for the C/N ratio (g·g^−1^) was calculated for all samples (Equation (2)). The C/N ratio connects the N content, an important macronutrient, as a positive indicator for leaf nutritional quality with the C content, an indicator for phenolics (quantitative defence compound), as a negative indicator for leaf palatability. According to the carbon-nutrient balance hypothesis [37], an increase in C/N ratio positively correlates with levels of defence compounds. For further nutritional analyses, the mean values were used for each forest layer per sample site.

(2) C/N ratio=C content (g)/N content (g)

The chlorophyll content of leaves correlates with leaf N content [38], because up to 75% of N content is located in chloroplasts [39]. Measurements of chlorophyll content index (CCI) were conducted with a CCM-200 plus Chlorophyll content meter (Opti-Sciences Inc., Hudson, NH, USA). The CCI increases with the chlorophyll content of leaves. Ten CCI values were taken directly in the field in June 2012 for each individual tree and forest layer at all sample sites. The mean values of chlorophyll were used of each forest layer per sample site for further analyses.

### 2.3. Herbivory Data

Arthropod herbivory was assessed with a visual inspection of adaxial and abaxial sides of leaf samples. Therefore, foliage material was defrosted and all leaves from two 30 cm long branches (starting at the tip of the branch) per tree individual and forest layer were surveyed (1799 understorey leaves, 2158 lower canopy leaves, and 2665 upper canopy leaves). All leaves were checked for all four classes of herbivore feeding traces (leaf-chewing, sap-sucking, leaf-mining, and gall-inducing). A species level identification for the feeding traces was reduced, because an unequivocal attribution of damage to a particular arthropod species, especially belonging to exophagous feeding guilds, is often impossible. Feeding traces were sorted into groups of homogeneous appearance and considered as recognizable taxonomic units (RTUs). Ecological research often uses RTUs for indices of abundances [40,41,42,43]. Overall, 15 feeding traces were identified (Appendix A) and voucher specimens were stored at the Department of Biology, University of Hildesheim, Hildesheim, Germany.

For every detected feeding trace, the number of attacked leaves was counted and used as the percentage of the total amount of leaves per sample, representing the herbivore attack levels. For each feeding trace, the associated arthropod species, probably causing the feeding trace, was determined with identification databases and literature [44,45,46,47,48,49]. Most of the feeding traces were also analysed in a study written by Gossner et al. [50] and are in accordance with the associated arthropod herbivore species in this study. Larvae of endophagous arthropod species were found within the herbivore feeding traces on leaf samples. Additionally, insect samples were taken in the understorey, lower and upper canopy at all forests sites with a beating net for the identification of the probable exophagous insect herbivore species. Voucher specimens of *Orchestes fagi* Linnaeus (Coleoptera: Curculionidae), *Phyllobius argentatus* Linnaeus (Coleoptera: Curculionidae), *Fagocyba cruenta* Herrich-Schäffer (Hemiptera: Cicadellidae), and *Phyllaphis fagi* Linnaeus (Hemiptera: Callaphididae) were stored at the Department of Biology, University of Hildesheim, Hildesheim, Germany. Since about two thirds of the feeding traces belonged to endophagous leaf-mining and gall-inducing feeding guilds, their feeding traces were more suitable for the identification than those of exophagous feeding guilds. Due to a high specialization of endophagous arthropod herbivores by internal interactions with the host plant physiology, feeding traces were well distinguishable based on special differences in form and appearance of galls and mines.

### 2.4. Statistical Analyses

Eight feeding traces that occurred at all forest sites (Appendix B) were further investigated concerning their distribution along the vertical forest gradient. The selection included two leaf-chewing, one sap-sucking, one leaf-mining, and four gall-inducing feeding traces. Herbivory was regarded as herbivore attack level, measured as the numbers of leaves bearing the feeding trace (as percentage of the sample). The spatial distribution of oviposition can result in clumping of mines and galls. Single occasions of mine and gall clumping were averaged using the 20 sample sites to overcome the influence of clumping on the data set. Herbivore attack levels of all eight feeding traces were compared between juvenile and adult *F. sylvatica* along the vertical forest gradient. An adequate comparison of leaf herbivory between individual plants would necessarily rely on similar leaf sizes. Leaf size was higher for leaves in lower canopies compared to similar average values of leaves in the understorey and upper canopies (Appendix C). However, leaf size had no overall effect on herbivore attack levels (*F*_1478_ = 0.693, *p* = 0.405), neglecting an influence for the comparison of herbivore attack levels between different plant individuals. Statistical analyses for significant comparison were performed with the R Version 3.4.1 [51]. The statistical distribution of the data (microclimate, leaf traits, and herbivory) was assessed with the Shapiro-Wilk-test, which was necessary to select between ANOVA or Kruskal-Wallis tests for analyses of variance. Based on the nonparametric data for herbivore attack levels, significant comparisons were performed with Kruskal-Wallis and post-hoc-tests for all eight feeding traces on juvenile and adult *F. sylvatica* along the vertical forest gradient (Equations (3) and (4)):

(3) kruskal.test(herbivore attack level ~ forest layer)

(4) kruskalmc(herbivore attack level ~ forest layer)

With eight dependant variables for herbivory (number of feeding traces) and seven independent variables for microclimate and leaf traits (temperature, relative air humidity, SLA, Nand C content, C/N ratio, and chlorophyll content) multivariate statistics was firstly chosen for analysis. The aim was to illustrate the ecological and environmental (dis)similarities between the occurrence of feeding traces in terms of microclimate, leaf trait parameters, and plant ontogenetic stage along the vertical forest gradient. On the basis of nonparametric data for herbivore attack levels, the ordination was generated using non-metric multidimensional scaling (NMDS) [52]. Calculations were done with the R packages *vegan* [53] and *goeveg* [54] based on the Bray-Curtis dissimilarity. The full R script for calculating the NMDS is available in Appendix D. For constructing the ordination, the *dimcheckMDS* function was used for detecting the best dimensionality in NMDS. The *dimcheckMDS* function provided a diagnostic plot of stress values for six tested dimensions in NMDS (Appendix D), showing the decrease in ordination stress with an increase in the number of ordination dimensions. Based on the diagnostic plot, two dimensions were used for the ordination. The NMDS was calculated with data of herbivore attack levels for all eight feeding traces. Sample sites along the vertical forest gradient (corresponding to juvenile and adult *F. sylvatica*) were plotted onto the ordination. Arthropod herbivory was interpreted based on post-hoc correlations with microclimate (temperature and relative air humidity) and leaf trait parameters (SLA, N and C content, C/N ratio, and chlorophyll content). Significant parameters were fitted onto the biplot.

Effects of parameters on arthropod herbivory along the vertical forest gradient were determined with linear mixed models (LMM) and a following model selection. Calculations were done using the R libraries *lme4* for LMM [55] and *MuMIn* for the model selection [56]. Herbivore attack levels were either square-root transformed (small circles and whitish spots) or log-transformed (labyrinth, tubular mine, leaf edge gall, haired vein gall, pannose spot, and ovate gall), depending on the best reduction for skewed statistical distribution of the nonparametric data. All models contained the study site as a random effect. For the herbivore attack levels on *F*. *sylvatica* leaves caused by the eight feeding traces, model comparisons were conducted for effects of microclimate (temperature and relative air humidity), leaf traits (SLA, N and C content, and C/N ratio), and plant ontogenetic stage with a full model specification. The best models were selected based on the Bayesian Information Criterion (BIC) (Appendix E). Linear regressions for herbivore attack levels and the determining parameters of the best models, preferring single parameters, were calculated for all eight feeding traces.

## 3. Results

### 3.1. Herbivore Feeding Traces

Overall, 15 different feeding traces were identified for *F. sylvatica* (Table 1 and Appendix A). Identified feeding traces belonged to leaf-chewing (3), sap-sucking (2), leaf-mining (5), and gall-inducing (7) feeding guilds. Ubiquitous feeding traces, like small circles and whitish spots, were found on all while haired vein galls and pannose spots were found on almost all, juvenile and adult *F. sylvatica* sample site (Appendix B).

**Figure 2:**
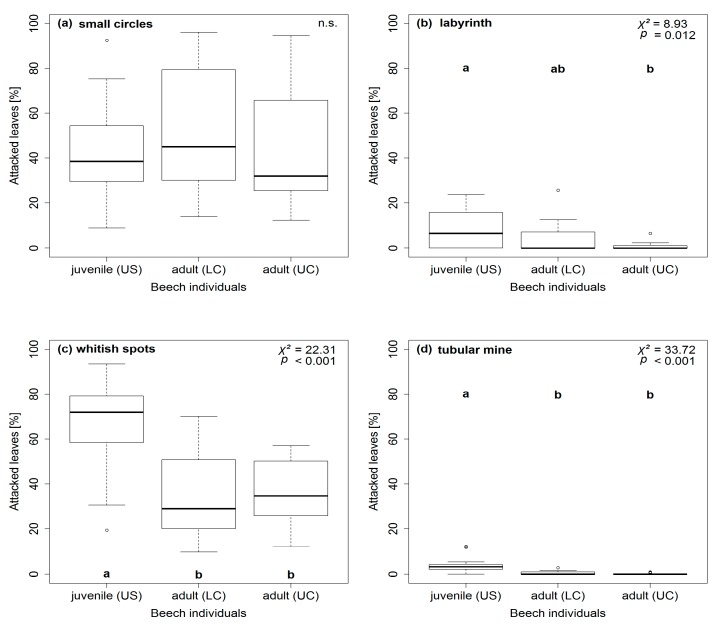
Distributions of herbivore attack levels for identified feeding traces along the vertical forest gradient. Percentages of attacked leaves on juvenile (US = understorey) and adult (LC = lower canopy, UC = upper canopy) *Fagus sylvatica* (*n* = 60) are presented for the leaf-chewing feeding guild (**a**) small circles; (**b**) labyrinth; the sap-sucking feeding guild (**c**) whitish spots; and the leaf-mining feeding guild (**d**) tubular mine. Boxplots are marked with lowercase letters indicating significant differences using Kruskal-Wallis and post-hoc test (*p* ≤ 0.05; df = 2) or with “n.s.” for non-significant differences.

**Figure 3:**
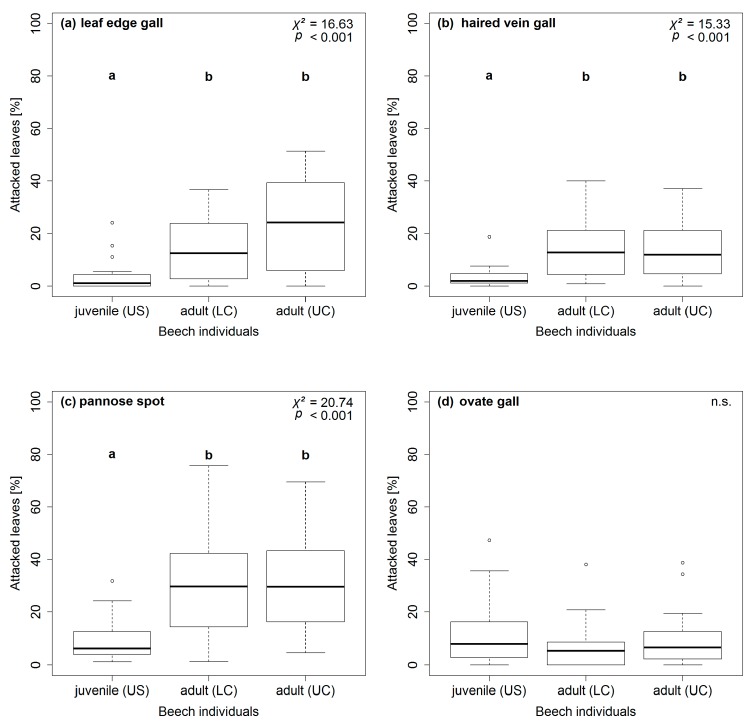
Distributions of herbivore attack levels for identified feeding traces along the vertical forest gradient. Percentages of attacked leaves on juvenile (US = understory) and adult (LC = lower canopy, UC = upper canopy) *Fagus sylvatica* (*n* = 60) are presented for the gall-inducing feeding guild (**a**) leaf edge gall; (**b**) haired vein gall; (**c**) pannose spot; and (**d**) ovate gall. Boxplots are marked with lowercase letters indicating significant differences using Kruskal-Wallis and post-hoc test (*p* ≤ 0.05; df = 2) or with “n.s.” for non-significant differences.

Along the vertical forest gradient, patterns of herbivore attack levels differed between the eight feeding traces (Figure 2 and Figure 3). On the one hand, herbivore attack levels on *F. sylvatica* leaves were higher on juveniles, compared to adults, for labyrinths, whitish spots, and tubular mines (Figure 2b–d). On the other hand, *F. sylvatica* leaves of adults were more often attacked than leaves of juveniles by leaf edge galls, haired vein galls, and pannose spots (Figure 3a–c). Herbivore attack levels for small circles and whitish spots did not vary significantly between the two ontogenetic stages of *F. sylvatica* (Figure 2a and Figure 3d).

Overall, herbivore attack levels on leaves varied between different feeding traces (Table 2). Herbivore attack levels on *F. sylvatica* leaves were highest for small circles (leaf-chewing) and whitish spots (sap-sucking), intermediate for all galls, and low for labyrinths (leaf-chewing) and tubular mines. Highest herbivore attack levels on juvenile *F. sylvatica* were also found for small circles and whitish spots, with percentages much higher than all other feeding traces. On adult *F. sylvatica*, herbivore attack levels of pannose spots between veins (galls) reached the high magnitude of small circles and whitish spots. Feeding traces of labyrinths and tubular mines were rarely seen on leaves of adult *F. sylvatica*.

### 3.2. Feeding Trace Composition

**Figure 4:**
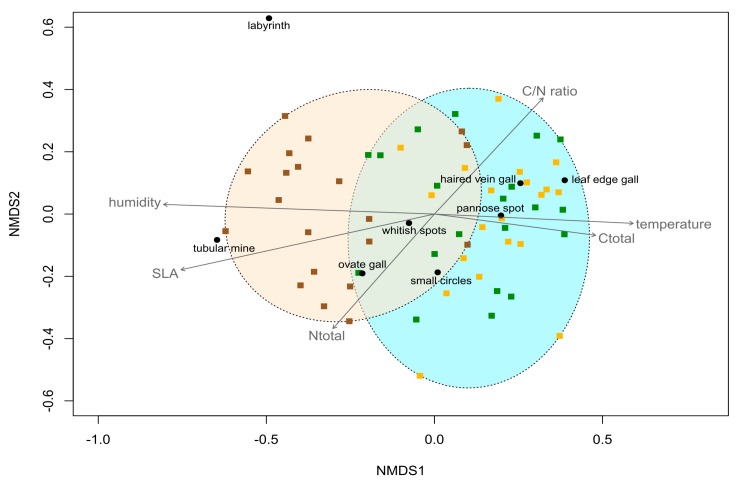
Ordination of samples and arthropod herbivore attack levels in a biplot with non-metric multidimensional scaling (NMDS). Each point of a sample site represents the composition of herbivore attack levels. Herbivore attack levels caused by identified feeding traces (black circles) are orientated along microclimate (temperature, humidity) and leaf traits (SLA, Ctotal, Ntotal, C/N ratio) parameter axes (post-hoc correlations). Sample sites along the vertical forest gradient are presented for the understorey (brown squares), lower canopy (green squares), and upper canopy (yellow squares), representing juvenile (beige ellipse) and adult (blue ellipse) *Fagus sylvatica*. Non-metric multidimensional scaling is based on Bray-Curtis dissimilarity (stress = 0.187 (usable)). Temperature (*R*^2^ = 0.249, *p* = 0.002), humidity (*R*^2^ = 0.461, *p* = 0.001), SLA (*R*^2^ = 0.426, *p* = 0.001), Ctotal (*R*^2^ = 0.167, *p* = 0.007), Ntotal (*R*^2^ = 0.161, *p* = 0.009), and C/N ratio (*R*^2^ = 0.173, *p* = 0.005) parameters represent significant axes for the NMDS ordination. Temperature = air temperature; humidity = relative air humidity; SLA = specific leaf area; Ctotal = leaf C content; Ntotal = leaf N content.

Herbivore attack levels of the eight feeding traces, as well as forest layer sample sites (representing juvenile and adult *F. sylvatica*), were ordinated in a biplot with NMDS along the environmental and leaf trait parameter axes (Figure 4). The stress value was 0.187 and goodness of NMDS was determined with the category “usable” (stress value < 0.20) following the guidelines for acceptable stress values [57]. A Shepard diagram is available in Appendix D. Temperature, relative air humidity, SLA, leaf C content, leaf N content, and C/N ratio axes were significant parameters for the NMDS ordination (post-hoc correlations). The chlorophyll parameter was deleted due to non-significance (*R*^2^ = 0.054, *p* = 0.234). Data for microclimate and leaf traits of juvenile and adult *F. sylvatica* along the vertical forest gradient are available in Appendix C. The NMDS showed a complete overlap of feeding trace compositions for lower and upper canopy leaves, and also a slight overlap for juvenile and adult *F. sylvatica*. Along environmental and leaf trait parameter axes, the orientation varied between the three forest layers. Sample sites with juveniles of *F. sylvatica* were orientated towards increasing air humidity, SLA and, to a lesser degree, towards leaf N content. In contrast to juveniles, adult *F. sylvatica* exhibited an orientation towards increasing temperature, leaf C content, and C/N ratio. Lowest distances for the feeding traces existed between leaf edge gall, haired vein gall, and pannose spot (ordinated within adults), as well as between small circles, whitish spots, and ovate gall (ordinated within the overlap of juveniles and adults). Labyrinth and tubular mine feeding traces showed the greatest distances to the other feeding traces.

### 3.3. Effects of Microclimate, Leaf Traits, and Plant Ontogenetic Stage

Based on the BIC, herbivore attack levels of the eight feeding traces on juvenile and adult *F*. *sylvatica* along the vertical forest gradient were best explained by different parameters (Table 3, Appendix E). The best predicting parameters were relative air humidity, N content, C/N ratio, and plant ontogenetic stage. Linear regressions based on the best predictors for herbivore attack levels on *F*. *sylvatica* showed significant effects, except for small circles and ovate galls (Table 3). The plant ontogenetic parameter significantly explained all other feeding traces. Effects of C/N ratio were positive on whitish spots and tubular mine, or negative on leaf edge gall, haired vein gall, and pannose spot (Appendix E). In contrast, effects of N content were negative on tubular mine or positive on leaf edge gall, haired vein gall, and pannose spot.

## 4. Discussion

This study revealed varying herbivore attack levels between (overall values) and within (values along the vertical forest gradient) different feeding traces. A majority of feeding traces occupied different layers in forest stands, with distinct preferences for juvenile or adult trees of *Fagus sylvatica* (European beech). In addition, patterns of herbivore attack levels also differed within feeding guilds. Highest herbivore attack levels were found for small circles and whitish spots (Table 2), both belonging to the exophagous feeding guild. Gall-inducing feeding traces revealed herbivore attack levels lying in between the highest and lowest herbivore attack levels. Lowest herbivore attack levels were caused by one exophagous and endophagous feeding trace (labyrinth and tubular mine, respectively). These findings are in accordance with other studies. For *Acer pseudoplatanus* (Sycamore maple), levels of herbivory (proportion of attacked leaves) for sap-sucking, leaf-mining, and gall-inducing feeding guilds show the same differences [31], comparable to the values on *F. sylvatica* in this study. Leaf-mining often forms only a minor component of herbivore damage due to low levels of abundance [58]. Overall, differences in herbivory levels are based on the mobile ability of exophagous insects to exploit many leaves on various plants. In contrast, single individuals of arthropod herbivore species belonging to the endophagous feeding guild are naturally restricted to one leaf of a plant individual.

In this study, six out of eight feeding traces showed differences along the vertical forest gradient concerning herbivore attack (Figure 2 and Figure 3). However, levels of herbivore attack only differed significantly between juvenile and adult *F. sylvatica*, but not within the canopy. On the one hand, three out of four feeding traces of the gall-inducing feeding guild (probably acari) showed increased herbivore attack levels on adult *F. sylvatica* compared to juveniles. On the other hand, herbivore attack levels of labyrinth and whitish spots (leaf-chewing and sap-sucking feeding guild, respectively) were higher on juveniles, compared to adult *F. sylvatica*. The distinct patterns are the same for gall-inducing and sap-sucking feeding traces on leaves of *A. pseudoplatanus* [31]. Differences along the whole vertical gradient, even between lower and upper canopies, are present for microclimate (temperature and relative air humidity) and leaf traits (SLA and C content; N content and C/N ratio only differ within the canopy) for *F. sylvatica* (Appendix C). Therefore, distribution patterns of herbivore attack levels in the understory and canopy do not seem to be driven by microclimate and leaf trait parameters. Instead, the plant ontogenetic stage had a stronger effect on herbivore attack levels than microclimate or leaf traits (Table 3). This leads to the predominate role of plant ontogeny, not the environment, affecting herbivory on *F. sylvatica* along the vertical forest gradient. The remaining question is the underlying cause behind the plant ontogenetic effect on preferences of arthropod herbivores for juvenile or adult *F. sylvatica*.

The ontogenetic variation of leaf toughness [10,17] is linked to the accumulation of phenolic compounds and lignifications of leaf tissues [59]. Furthermore, phenolic compounds are indicated by C content because mechanical or chemical defences are often carbon-based [60,61]. Despite the absence of differences in plant defences between juvenile and mature woody species [22], leaves of juvenile and adult *F. sylvatica* in this study differed in toughness (indicated by SLA) and C content. Since these differences were also present between the lower and upper canopy, a potential effect on herbivory could only be applied to a non-linear relationship. In this case, values for leaf C content and toughness would cross a threshold from juvenile to adult *F. sylvatica* individuals that could cause the existing herbivory shift. Another possible reason for distinct preferences of some feeding guilds for juvenile or mature plants can also be enemy-free space [13]. Predation by birds [62], predatory wasps [63], and parasitoids [64] is higher in mature, compared to juvenile, trees. According to the hypothesis of harsh environment, herbivory of galls is higher in xeric, compared to mesic, habitats, which is explained by different mortality rates through parasitoids and fungi [65]. The lower top-down control by parasitoids also affects gall patterns along the vertical gradient in mesic tropical rainforests [66]. Testing the hypothesis of a harsh environment as an example of an enemy-free space along the vertical gradient of temperate forests would require counting the galls and separating them into dead and living galls. This represents a different measure of herbivory than that used in this study and could lead to different results for herbivory levels.

Feeding traces of small circles (leaf-chewing) did not show significantly-distinct herbivore attack levels along the vertical forest gradient in this study. However, using a different measure for herbivory caused by the leaf-chewing feeding guild, quantifying the missing leaf area, leads to an increased herbivory in the understorey compared to upper canopies [25]. This pattern seems to be driven by indirect effects of environmental conditions, causing leaves in the understory to be more palatable for leaf-chewing insect herbivores. The natural movement of the leaf-chewing weevil, *Phyllobius argentatus*, which is more active in the understory, from one tree species to another, depends on the palatability of the leaves [67]. Patterns of leaf palatability for *F. sylvatica* along the vertical forest gradient can be adapted to general differences between young and mature leaves. Peak densities of exophagous feeding guilds are associated with new leaf samples on most plant species [68]. Many structural features develop with increasing leaf age that makes feeding on mature leaves more difficult for exophagous herbivore insects [28]. Features include tougher leaves and higher defence compounds that are also caused in *F. sylvatica* leaves by environmental conditions (light, microclimate, and water stress) along the vertical forest gradient.

## 5. Conclusions

Despite the overall high levels of herbivory caused by exophagous feeding traces, patterns of herbivore attack levels vary within different feeding traces between juvenile and adult *F. sylvatica*. In addition, levels and distribution patterns of herbivore attacks are even distinct between different feeding traces within one feeding guild. Therefore, general conclusions on herbivory patterns can hardly be drawn at the species level, but rather are possible as averages for whole feeding guilds. This would be in accordance with the assumption of Kozlov et al. [69] that the type of damage is more important than the identity of the causer from the plant’s perspective. Furthermore, the great diversity of arthropod herbivores and feeding guilds cannot lead to one general plant ontogenetic pattern. This study presents findings for the importance to differentiate between distinct feeding guilds of insect herbivores for research about plant ontogenetic effects on herbivory. The underlying causes for ontogenetic preferences need to be addressed in future studies. Changes in leaf traits affecting herbivory are found between the ontogenetic stages of juvenile and adult *F. sylvatica* along the vertical forest gradient. However, if these leaf traits represent underlying causes for plant ontogenetic preferences of insect herbivores, relationships cannot be linear, since important leaf traits also change between lower and upper canopies of adult *F. sylvatica*.

## Figures and Tables

**Table 1:** List of 15 identified arthropod herbivore feeding traces (recognizable taxonomic unit = RTU) on leaves of *Fagus sylvatica*. Presented are feeding traces for (a) exophagous and (b) endophagous feeding guilds. Images of all feeding traces are available in Appendix A.

Feeding Trace ^1^	Description	Guild ^2^	Leaf Side ^3^	Probable Arthropod Species ^1^
**(a) Exophagous:**				
windows	scraping damage on leaf surface	ch		*Diurnea fagella* (Denis and Schiffermüller, 1775)
**small circles**	missing leaf area as small circles	ch		*Orchestes fagi* (Linnaeus, 1758)
**labyrinth**	missing leaf area in form of labyrinths	ch		*Phyllobius argentatus* (Linnaeus, 1758)
**whitish spots**	leaf flecked with whitish spots	s	ADS	*Fagocyba cruenta* (Herrich-Schäffer, 1838)
wax wool	waxed threads on leaf surface	s	ABS	*Phyllaphis fagi* (Linnaeus, 1767)
**(b) Endophagous:**				
**tubular mine**	tubular shaped mine between lateral veins	m	ABS	*Phyllonorycter maestingella* (Müller, 1764)
oval mine	oval shaped mine between lateral veins	m	ABS	*Phyllonorycter messaniella* (Zeller, 1846)
line crossing veins	wide corridor mine crossing lateral veins	m		*Stigmella hemargyrella* (Kollar, 1832)
line between veins	zigzag mine between lateral veins	m		*Stigmella tityrella* (Stainton, 1854)
**leaf edge gall**	gall causing rolled-up leaf edges	g		*Acalitus stenaspis* (Nalepa, 1891)
**haired vein gall**	haired gall along lateral leaf veins	g	ADS	*Aceria nervisequa* (Canestrini, 1891)
**pannose spot**	pannose spot between lateral veins	g	ABS	*Aceria nervisequa faginea* (Nalepa, n.d.)
haired brownish gall	cylindrical, haired brownish gall	g	ADS	*Hartigiola annulipes* (Hartig, 1839)
**ovate gall**	ovate, acuminated gall (green to red)	g	ADS	*Mikiola fagi* (Hartig, 1839)
pleated gall	swollen, pleated leaf tissue forming a pouch	g	ADS	*Phegomyia fagicola* (Kieffer, 1901)

^1^ Feeding traces were identified as RTUs and assigned to the probably responsible arthropod herbivore species using identification databases and literature [44,45,46,47,48,49]. Feeding traces in boldface: distributions of herbivory were analysed along the vertical forest gradient. ^2^ ch = leaf-chewing, s = sap-sucking, m = leaf-mining, g = gall-inducing. ^3^ ADS = adaxial side, ABS = abaxial side.

**Table 2:** Herbivore attack levels (percentage of attacked leaves) of feeding traces on leaves of *Fagus sylvatica* (*n* = 60). Comparisons are shown for leaves along the vertical forest gradient, as well as for leaves of juvenile and adult *F. sylvatica*. Values represent the median and interquartile ranges (IQR = first quartile, third quartile). Boxplots with lowercase letters indicate significant differences for overall attacks and the comparison within juvenile and adult *F. sylvatica* using Kruskal-Wallis and post-hoc test (*p* ≤ 0.05; df = 7).

Feeding Guild	Feeding Trace	Attack Leaves (%)		
		Overall	Juvenile Beech	Adult Beech
leaf-chewing	small circles	36 (29,65) ^a^	39 (30,54) ^a^	35 (28,72) ^a^
	labyrinth	0 (0,7) ^b,c^	6 (0,13) ^b^	0 (0,2) ^b,d^
sap-sucking	whitish spots	41 (27,60) ^a^	72 (59,78) ^a^	32 (23,49) ^a^
leaf-mining	tubular mine	0 (0,2) ^c^	3 (2,4) ^b^	0 (0,0) ^b^
gall-inducing	leaf edge gall	9 (1,24) ^d,e^	1 (0,4) ^b^	19 (3,32) ^c,d,e^
	haired vein gall	7 (2,16) ^d^	2 (1,4) ^b^	12 (5,21) ^e^
	pannose spot	18 (7,35) ^e^	6 (4,12) ^b^	30 (15,42) ^a,c^
	ovate gall	6 (2,13) ^b,d^	8 (3,15) ^b^	13 (4,21) ^d,e^

**Table 3:** Effects of microclimate, leaf trait parameters, and plant ontogenetic stage on arthropod herbivore attack levels (linear regressions) based on the best calculated models compared with the Bayesian Information Criterion (ΔBIC = 0–2) (Appendix E). The preference was set on models with the lowest number of parameters, resulting in choices of single parameters or the combination of two parameters (without interaction). The effect of the plant ontogenetic stage was tested for all exophagous and endophagous feeding traces. Herbivore attack levels on *F. sylvatica* (*n* = 57) were square-root transformed (small circles and whitish spots) or log-transformed (labyrinth, tubular mine, leaf edge gall, haired vein gall, pannose spot, and ovate gall), depending on the best reduction for skewed statistical distribution of the nonparametric data.

Model	d.f.		*F*-Values ^1,2^							
Parameters			Exophagous			Endophagous				
	n.	d.	Small Circles	Labyrinth	Whitish Spots	Tubular Mine	Leaf Edge Gall	Haired Vein Gall	Pannose Spot	Ovate Gall
humidity	1	55								**1.29**
N content	1	55	**0.98**							
N content + stage	2	54				20.50 ***	16.99 ***	14.62 ***	18.15 ***	
CN + stage	2	54			17.72 ***	21.08 ***	16.31 ***		17.25 ***	
stage	1	55	0.19	9.79 **	30.85 ***	37.25 ***	17.96 ***	18.64 ***	27.56 ***	**1.33**

^1^ *F*-values in boldface: best models based on the BIC (ΔBIC = 0–2); ^2^ Results of analyses of variance: * = *p* < 0.05; ** = *p* < 0.01; *** = *p* < 0.001; n. = numerator; d. = denominator; humidity = relative air humidity; N content = leaf nitrogen content; stage = plant ontogenetic stage; CN = C/N ratio.

**Figure A1:**
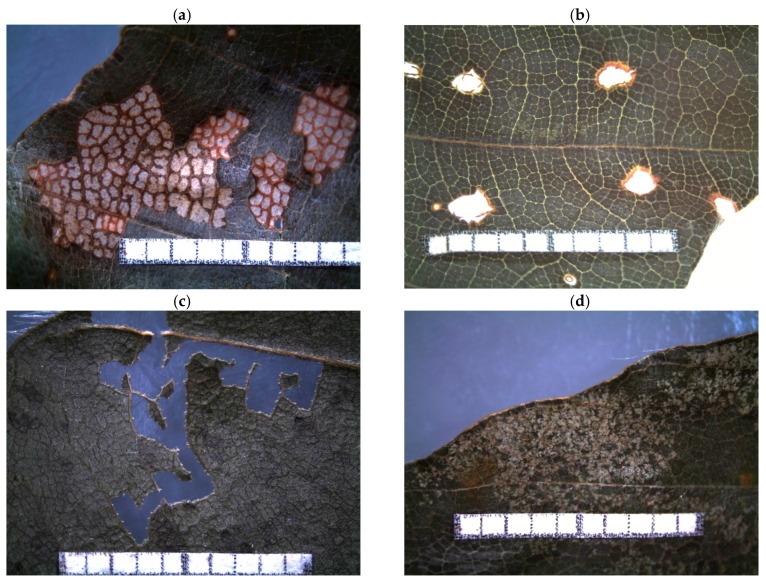

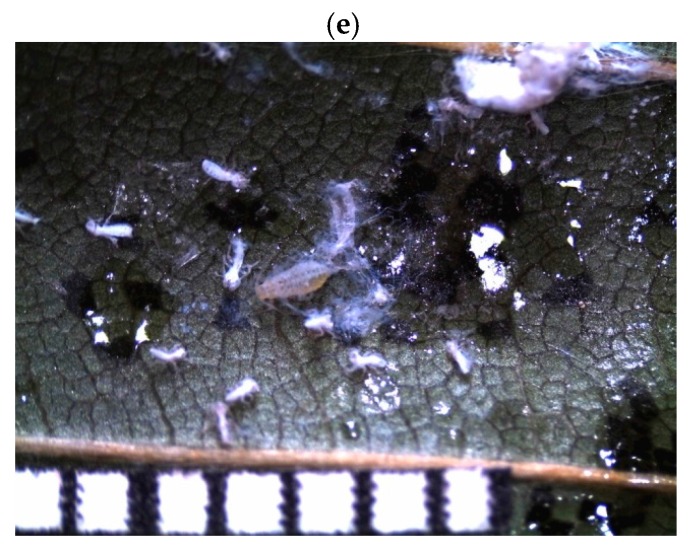
Feeding traces (Recognizable Taxonomic Units) of leaf-chewers and sap-suckers (exophagous feeding guilds) on *Fagus sylvatica* leaves. (**a**) Windows (probably *Diurnea flagella*, ADS); (**b**) small circles (probably *Orchestes fagi*, ADS); (**c**) labyrinth (probably *Phyllobius argentatus*, ADS); (**d**) whitish spots (probably *Fagocyba cruenta,* ADS); (**e**) wax wool (probably *Phyllaphis fagi*, ABS). Images were taken with an integrated digital camera of a stereo microscope (LEICA EZ4 D, Wetzlar, Germany). Little square equals 1×1 mm, ADS = adaxial side, ABS = abaxial side.

**Figure A2:**
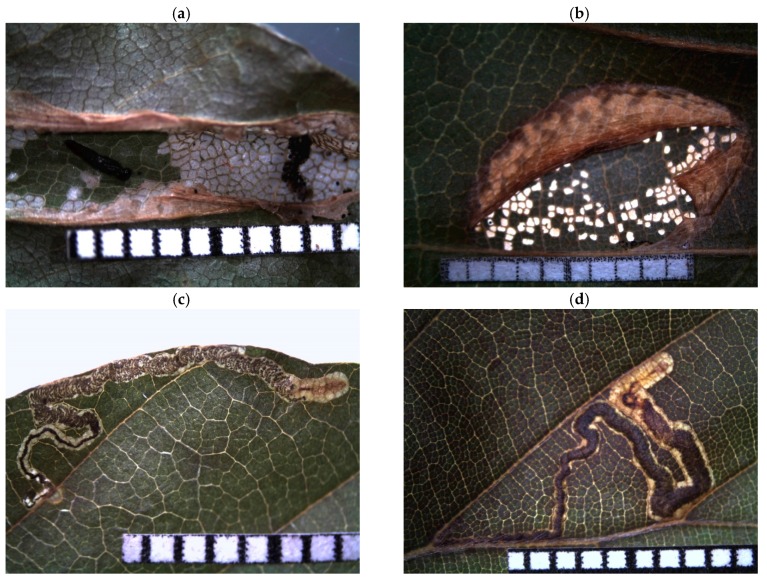
Feeding traces (Recognizable Taxonomic Units) of miners (endophagous feeding guild) on *Fagus sylvatica* leaves. (**a**) Tubular mine (probably *Phyllonorycter maestingella*, ABS); (**b**) oval mine (probably *Phyllonorycter messaniella*, ABS); (**c**) line crossing lateral veins (probably *Stigmella hemargyrella*, ADS); (**d**) line between lateral veins (probably *Stigmella tityrella*, ADS). Images were taken with an integrated digital camera of a stereo microscope (LEICA EZ4 D, Wetzlar, Germany). Little square equals 1×1 mm, ADS = adaxial side, ABS = abaxial side.

**Figure A3:**
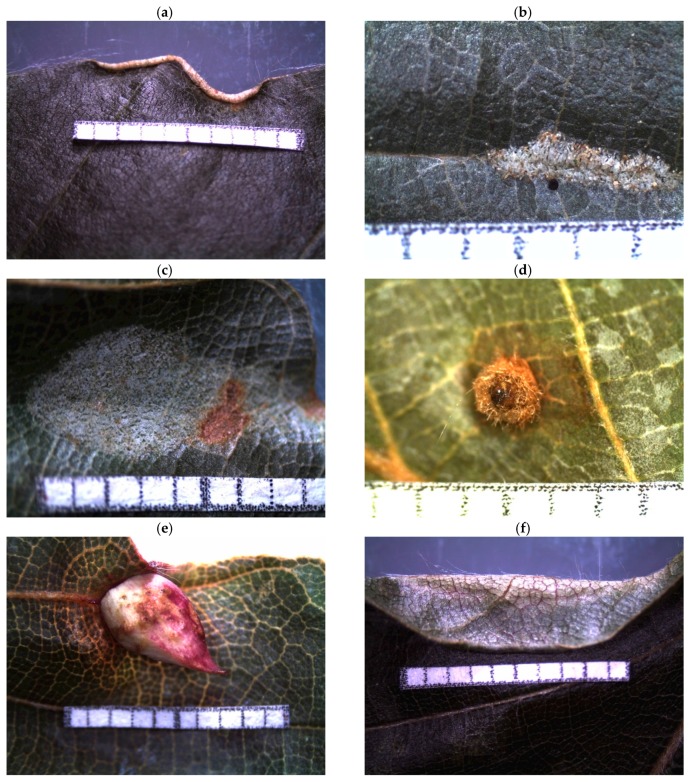
Feeding traces (Recognizable Taxonomic Units) of gall-inducers (endophagous feeding guild) on *Fagus sylvatica* leaves. (**a**) Rolled-up leaf edge (probably *Acalitus stenaspis*, ADS); (**b**) haired gall on leaf veins (probably *Aceria nervisequa*, ADS); (**c**) pannose spot between veins (probably *Aceria nervisequa faginea*, ABS); (**d**) haired brownish gall (probably *Hartigiola annulipes*, ADS); (**e**) ovate, acuminated gall (probably *Mikiola fagi*, ADS); (**f**) pleated gall (probably *Phegomyia fagicola*, ADS). Images were taken with an integrated digital camera of a stereo microscope (LEICA EZ4 D, Wetzlar, Germany). Little square equals 1×1 mm, ADS = adaxial side, ABS = abaxial side.

**Figure A4:**
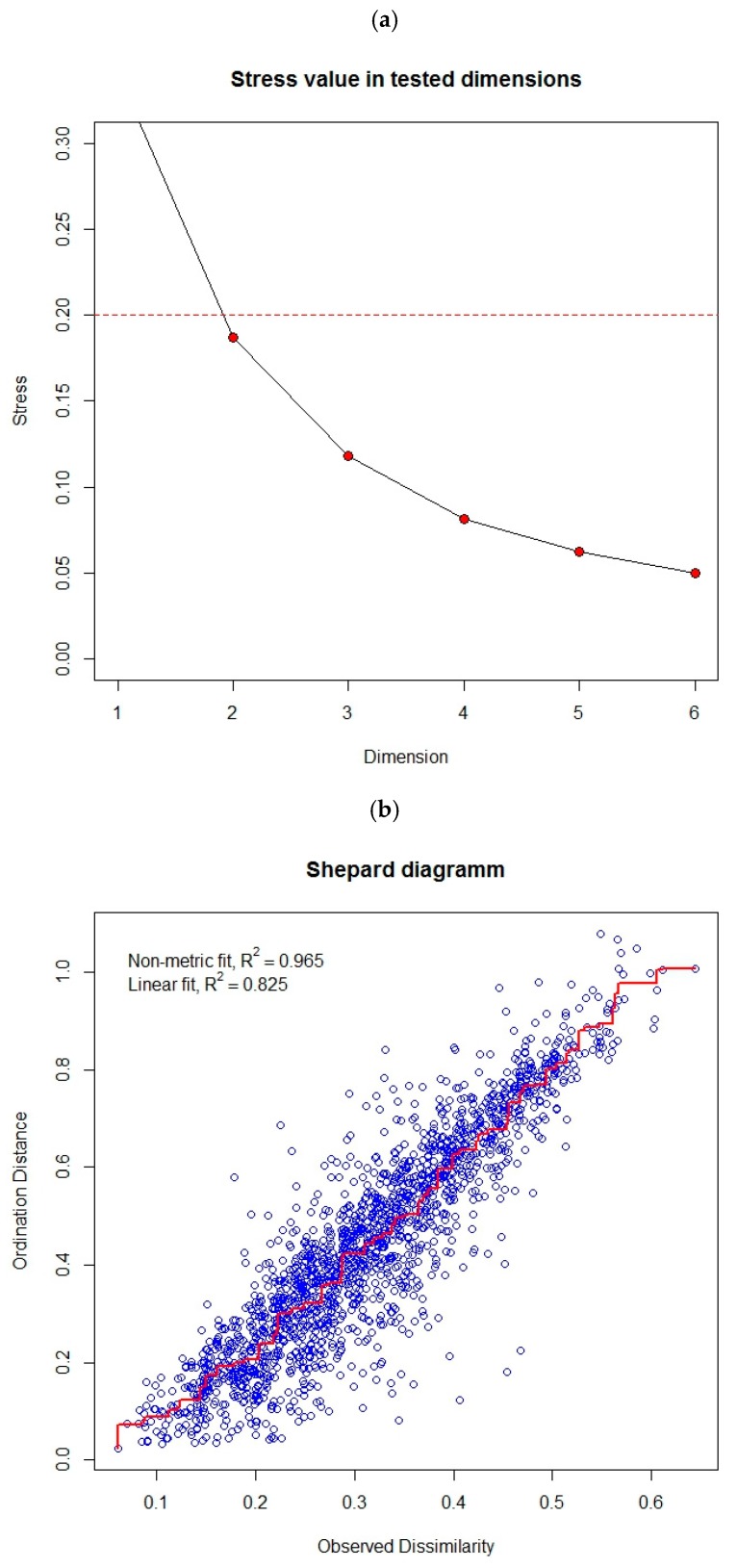
(**a**) Plot of stress indicating 2 (first point under the dotted line) as the best dimensionality for the ordination with non-metric multidimensional scaling (NMDS) and (**b**) Shepard diagram for the NMDS.

## Appendix A

Herbivore feeding traces (recognizable taxonomic units) of exophagous (leaf-chewing and sap-sucking), and endophagous (leaf-mining, and gall-inducing) feeding guilds.

## Appendix B

**Table A1:** Presence (+) and absence of feeding trace morphospecies on leaves of juvenile and adult *Fagus sylvatica* (European beech) individuals at all sample sites in the understorey and canopies, respectively. Data are based on feeding trace morphospecies identified on collected leaf samples.

Site	Beech	Leaf-Chewing		Sap-Sucking	Leaf-Mining	Gall-Inducing			
	Individuals	Small Circles	Labyrinth	Whitish Spots	Tubular Mine	Leaf Edge Gall	Haired Vein Gall	Pannose Spot	Ovate Gall
WB-N	juvenile (US)	+	+	+	+			+	+
	adult (LC)	+		+		+	+	+	+
	adult (UC)	+		+		+	+	+	+
WB-S	juvenile (US)	+	+	+	+		+	+	+
	adult (LC)	+		+	+	+	+	+	+
	adult (UC)	+		+		+	+	+	+
TE-N	juvenile (US)	+	+	+	+	+	+	+	+
	adult (LC)	+	+	+		+	+	+	+
	adult (UC)	+	+	+	+	+	+	+	+
TE-S	juvenile (US)	+	+	+	+	+	+	+	+
	adult (LC)	+	+	+	+	+	+	+	+
	adult (UC)	+		+		+	+	+	+
KBVB-N	juvenile (us)	+		+	+	+	+	+	+
	adult (lc)	+		+	+	+	+	+	+
	adult (uc)	+		+		+	+	+	+
KBVB-S	juvenile (US)	+	+	+	+	+	+	+	+
	adult (LC)	+		+		+	+	+	+
	adult (UC)	+		+		+	+	+	+
SS-N	juvenile (US)	+	+	+	+	+	+	+	+
	adult (LC)	+		+		+	+	+	
	adult (UC)	+		+	+	+	+	+	+
SS-S	juvenile (US)	+		+	+	+	+	+	+
	adult (LC)	+		+		+	+	+	+
	adult (UC)	+		+		+	+	+	+
HGB-N	juvenile (US)	+		+	+		+	+	+
	adult (LC)	+		+			+	+	+
	adult (UC)	+		+			+	+	+
HGB-S	juvenile (US)	+	+	+	+	+	+	+	+
	adult (LC)	+		+	+	+	+	+	+
	adult (UC)	+		+			+		+
BB-N	juvenile (US)	+	+	+	+			+	+
	adult (LC)	+		+		+	+	+	+
	adult (UC)	+		+		+	+	+	+
BB-S	juvenile (US)	+	+	+	+	+	+	+	+
	adult (LC)	+	+	+		+	+	+	+
	adult (UC)	+	+	+		+	+	+	+
HB-N	juvenile (US)	+	+	+	+		+	+	+
	adult (LC)	+	+	+		+	+	+	+
	adult (UC)	+	+	+		+	+	+	+
HB-S	juvenile (US)	+		+		+	+	+	+
	adult (LC)	+		+	+	+	+	+	
	adult (UC)	+		+		+	+	+	+
FK-N	juvenile (US)	+	+	+	+		+	+	
	adult (LC)	+	+	+		+	+	+	
	adult (UC)	+	+	+		+	+	+	
FK-S	juvenile (US)	+	+	+		+	+	+	
	adult (LC)	+	+	+		+	+	+	
	adult (UC)	+	+	+		+	+	+	+
HDB-N	juvenile (US)	+	+	+	+	+	+	+	
	adult (LC)	+		+		+	+	+	
	adult (UC)	+		+	+	+	+	+	+
HDB-S	juvenile (US)	+		+	+	+	+	+	
	adult (LC)	+		+	+	+	+	+	
	adult (UC)	+		+		+	+		
EL-N	juvenile (US)	+	+	+	+			+	+
	adult (LC)	+	+	+			+	+	+
	adult (UC)	+	+	+		+	+	+	+
EL-S	juvenile (US)	+		+	+	+	+	+	+
	adult (LC)	+	+	+	+	+	+	+	+
	adult (UC)	+	+	+		+	+	+	+

Forest sites: WB = Winkelberg, TE = Tiefentals Ebene, KBVB = Klingenberg/Vaaker Berg, SS = Schieferstein, HGB = Heiligenberg, BB = Bocksbühl, HB = Hubenberg, FK = Feuerkuppe, HDB = Heidelberg, EL = Eichleite, N = north exposition, S = south exposition. Forest layers: US = understorey, LC = lower canopy, UC = upper canopy.

## Appendix C

**Table A2:** Microclimate and leaf trait parameters of juvenile and adult *Fagus sylvatica* individuals along the vertical forest gradient. Microclimatic conditions for understory (US, *n* = 20), lower (LC, *n* = 20) and upper canopy (UC, *n* = 17) are represented by temperature and relative air humidity. Leaf trait parameters of *F. sylvatica* (US: *n* = 20; LC: *n* = 20; UC: *n* = 20) are represented by specific leaf area (SLA), total leaf carbon content (C), total leaf nitrogen content (N), C/N ratio, and chlorophyll content (chlorophyll). Presented are the median and the interquartile range (IQR = first quartile, third quartile).

Parameter	Beech Individual		
	Juvenile (US)	Adult (LC)	Adult (UC)
Microclimate:			
temperature (°C) ^1^	17.9 (17.6,18.2) ^a^	18.8 (18.6,19.1) ^b^	19.9 (19.6,20.6) ^c^
relative air humidity (%) ^2^	83 (80,86) ^a^	74 (72,76) ^b^	69 (68,70) ^c^
Leaf traits:			
SLA (m^2^ kg^−1^) ^2^	38 (35,42) ^a^	28 (24,33) ^b^	16 (15,18) ^c^
leaf area (cm^2^) ^2^	23 (21,24) ^a^	30 (23,34) ^b^	20 (19,23) ^a^
N (mg g^−1^) ^1^	22 (21,24) ^a^	23 (22,24) ^a^	21 (20,22) ^b^
C (mg g^−1^) ^1^	475 (472,748) ^a^	478 (476,480) ^b^	483 (481,486) ^c^
C/N ratio (g g^−1^) ^2^	21.3 (19.8,22.1) ^a^	21.0 (19.7,22.2) ^a^	23.4 (22.5,24.4) ^b^
chlorophyll (CCI) ^1^	13.8 (12.6,14.3) ^a^	13.5 (13.1,15.6) ^a^	13.2 (12.0,14.2) ^a^

Lowercase letters indicate significant differences of parameters between the forest layers using (1) ANOVA and Tukey's HSD (*p* ≤ 0.05; df = 2) or (2) Kruskal-Wallis and post-hoc test (*p* ≤ 0.05; df = 2). CCI = chlorophyll content index.

## Appendix D

R script for the ordination of sample sites and arthropod herbivore attack levels in a biplot with non-metric multidimensional scaling (NMDS) [52]. Calculations were done with R packages vegan [53] and goeveg [54].

**R codes:**

# detecting the best dimensionality in NMDS; the function provides a diagnostic plot of stress values for six tested dimensions in NMDS (see Figure A4a)

**dimcheckMDS(species, distance = “bray”, k = 6, trymax = 40, autotransform= TRUE)**

# calculation of NMDS with two dimensions; goodness of fit is indicated by the stress value; stress value 0.187 is determined with the category “usable” (stress value < 0.20) following the guidelines for acceptable stress values [57].

**nmds2 <- metaMDS(species, k = 2)**

**nmds2**

Call: metaMDS(comm = species, k = 2)
global Multidimensional Scaling using monoMDS
Data:  wisconsin(sqrt(species))
Distance: bray
Dimensions: 2
Stress:  0.1870401
Stress type 1, weak ties
Two convergent solutions found after 20 tries
Scaling: centring, PC rotation, halfchange scaling
Species: expanded scores based on ‘wisconsin(sqrt(species))

# plotting a Shepard diagram (see Figure A4b) showing the original pairwise distances (based on the Bray-Curtis dissimilarity) and the new distances of the ordination (based on ranks)

**stressplot(nmds2, main = “Shepard diagramm”)**

# fitting all environmental and leaf trait parameters onto the ordination as *post-hoc* correlations; selection of significant parameters (*p* < 0.05)

**variables_all <-envfit(nmds2, variables[6:12], choices=c(1,2), na.rm = TRUE**

**variables_all**

  ***VECTORS
       NMDS1  NMDS2  r2  Pr(>r)
temp_dayav  0.99872 -0.05053    0.2489  0.001 ***
humid_dayav   -0.99926  0.03837   0.4612  0.001 ***
SLA     -0.97297 -0.23095   0.4257  0.001 ***
Ctotal      0.99002 -0.14094   0.1665  0.006 **
Ntotal      -0.63438 -0.77302    0.1605  0.008 **
C.N      0.65518  0.75547    0.1729  0.007 **
chloro      -0.87043 -0.49229  0.0544  0.234
Permutation: free
Number of permutations: 999
3 observations deleted due to missingness

# fitting significant parameters onto the ordination as *post-hoc* correlations

**varisigEZ<- envfit(nmds2, varisig[6:11], choices = c(1,2), na.rm = TRUE)**

**varisigEZ**

***VECTORS
        NMDS1  NMDS2  r2 Pr(>r)  
  temperature 0.99872 -0.05051 0.2489  0.001 ***
  humidity  -0.99926  0.03836 0.4612  0.001 ***
  SLA    -0.97296 -0.23095 0.4257  0.001 ***
  C     0.99002 -0.14094 0.1665   0.007 **
  N     -0.63438 -0.77302 0.1605  0.009 **
  C.N    0.65518  0.75548 0.1729  0.005 **
  Permutation: free
  Number of permutations: 999
  3 observations deleted due to missingness

# creating the biplot graphic

**plot(nmds2, display=“species”, type = “n”, xlim=c(-1,0.8), ylim=c(-0.6,0.6))**

**ordiellipse(nmds2, groups = (variables$stage), display = “sites”, kind = (“ehull”), col=c(“cadetblue1”,“bisque1”), draw = c(“polygon”), lty=3)**

**points(nmds2, display=“sites”, pch=15, col=c(“tan4”,“forestgreen”,“darkgoldenrod1”)[(variables$layer)])**

**points(nmds2, display=“species”, pch=16)**

**plot(variablesEZ, col = “dimgray”, labels=c(“temperature”, “humidity”, “SLA”, “Ctotal”, “Ntotal”, “C/N ratio”))**

**ordilabel(nmds2, display=“species”, labels=c(“small circles”, “labyrinth”, “tubular mine”, “leaf edge gall”, “haired vein gall”, “pannose spot”, “ovate gall”, “whitish spots”), choices = c(1, 2), fill = NA, border = NA, col = “black”, xpd = TRUE)**

## Appendix E

Model comparisons for effects of microclimate, leaf traits and plant ontogenetic stage of Fagus sylvatica on herbivore attack levels caused by eight feeding traces along the vertical forest gradient. The models contain sample site as a random effect (1|site). Displayed are the twenty best models according to the Bayesian Information Criterion (BIC). Calculations were done using the R libraries lme4 [55] and MuMIn [56]. Different models have similar strength of evidence for ΔBIC = 0–2 and positive strength of evidence for ΔBIC = 2–6 against the model with the lowest BIC value [70]. The lowest BIC value implied either fewer explanatory variables, better fit, or both combined. Positive and negative values for the model variables indicate positive and negative effects on herbivore attack levels, respectively.

**R codes:**

# generic function dealing with NAs in the data frames; na.fail returns the object if it does not contain any missing values

**options(na.action = “na.fail”)**

# fiting the linear mixed-effects model (LMM) to data; REML is set FALSE because the model contains only one random effect (sample site)

**fullmod <-lmer(feeding_trace ~ stage + temp + humid + Ctotal + Ntotal + CN + SLA + (1|site), REML = FALSE)**

# generating a set of models with combinations (subsets) of fixed effect terms in the global model, with optional rules for model inclusion; dredge returns an object of class model selection, being a data frame with models' coefficients (or presence/NA for factors), *df* - number of parameters, log-likelihood, the information criterion value, delta-IC and *Akaike weight*. Models are ordered by the value of the information criterion specified by rank (lowest on top).

**dredge(update(fullmod), rank = “BIC”)**

**Small circles**

**Labyrinth**

**Whitish spots**

**Tubular mine**

**Leaf edge gall**

**Haired vein gall**

**Pannose spot**

**Ovate gall**
